# High-Grade Immature Gastric Teratoma in a 10-Day-Old Neonate: Diagnostic Challenges and Curative Surgical Management

**DOI:** 10.7759/cureus.83613

**Published:** 2025-05-06

**Authors:** Wajeeh Uddin, Mariam Aylan Alshamsi, Mohammed Alblooshi, Munir Ahmad, Masih Abdul Kader

**Affiliations:** 1 Pediatric Surgery, Al Jalila Children's Specialty Hospital, Dubai, ARE; 2 Medicine, Dubai Health, Dubai, ARE; 3 General Surgery, College of Medicine, University of Sharjah, Sharjah, ARE

**Keywords:** alpha fetoprotein monitoring, diagnostic imaging, immature teratoma (grade iii), neonatal gastric teratoma, surgical resection

## Abstract

Neonatal gastric teratomas are exceptionally rare germ cell tumors (<1% of pediatric teratomas) posing significant diagnostic and surgical challenges. Immature variants, particularly high‑grade tumors, can be locally aggressive yet typically have a favorable prognosis if completely resected. We report a 10‑day‑old male neonate (born at 36+1 weeks to a mother with gestational diabetes and pregnancy‑induced hypertension) presenting with a firm epigastric and left hypochondriac mass detected 24 hours after birth. Imaging revealed a mixed echogenic lesion containing fat and calcifications from the stomach’s lesser curvature, and serum alpha‑fetoprotein was markedly elevated for age. On day 12, an exploratory laparotomy achieved en bloc resection via partial gastrectomy with clear margins, despite dense adhesions to the left hepatic lobe and an intragastric component. Postoperatively, the patient experienced a right femoral artery thrombosis managed with anticoagulation and wound dehiscence addressed by local care and antibiotics. Histopathology confirmed a Grade III immature gastric teratoma without malignant germ cell elements; a splenule was also identified. An upper gastrointestinal contrast study on postoperative day 11 showed an intact repair, and feeds were successfully advanced. This case underscores the importance of suspecting gastric teratoma in neonates, interpreting age‑adjusted tumor markers carefully, and ensuring complete surgical excision. Vigilant perioperative management and long‑term surveillance with serial imaging and alpha‑fetoprotein monitoring remain paramount, particularly for high‑grade immature lesions.

## Introduction

Gastric teratoma is an exceedingly rare pediatric germ cell tumor, especially in the neonatal period. A teratoma, in particular, can contain a variety of tissue types, reflecting this broad differentiation potential. It accounts for less than 1% of all teratomas in infants and children, with only around one hundred cases reported in the literature [1,2]. Teratomas in pediatric patients most commonly occur in the sacrococcygeal region, followed by gonadal and other midline locations; in contrast, a teratoma arising from the stomach is an uncommon site [2]. Neonatal gastric teratomas predominantly affect male infants (the most reported cases have occurred in boys within the first few months of life) and they are considered the most common teratomas of the gastrointestinal tract despite their overall rarity [1,3].

Histologically, gastric teratomas are classified as either mature or immature, which has implications for clinical behavior. The majority of gastric teratomas are mature cystic tumors composed of well‑differentiated tissues from all three germ layers, and these generally behave in a benign fashion [3]. Immature gastric teratomas, containing embryonal or neuroectodermal elements, are much rarer and are of clinical concern due to their malignant potential; only a small number of these tumors have been documented, with occasional local invasion or, rarely, recurrence [1]. Malignant transformation within a gastric teratoma is exceptionally uncommon; however, the presence of a yolk sac tumor component has been linked to a higher risk of relapse, underscoring the importance of thorough histopathological evaluation [1].

Diagnosing a neonatal gastric teratoma can be challenging, as the clinical presentation and imaging findings are variable and these tumors may mimic other abdominal masses. Typically, affected infants present with a palpable abdominal mass or progressive abdominal distension; some may also experience feeding difficulty or non‑bilious vomiting due to compression of the stomach [2,4]. Rarely, gastric teratomas can cause gastrointestinal bleeding or respiratory compromise if the tumor ulcerates or grows large enough to elevate the diaphragm [1,4]. Imaging usually reveals a heterogeneous mass with mixed solid and cystic components, and the presence of calcification or fat within the lesion on ultrasound or computed tomography is a helpful clue suggesting a teratoma [4]. However, not all cases display these classic features, and a large cystic gastric teratoma may be misinterpreted as a more common cystic lesion (such as a lymphangioma), especially when internal hemorrhage obscures its fat or calcified components [4]. The differential diagnosis for a neonatal abdominal mass is broad, including more common neonatal tumors (e.g., neuroblastoma or hepatic neoplasms) as well as benign cystic lesions; thus, a gastric teratoma might not be immediately recognized [1]. Elevated alpha‑fetoprotein (AFP) levels may provide a diagnostic clue, but in neonates, the interpretation of AFP is limited by naturally high baseline levels, and definitive diagnosis rests on surgical resection with histopathological examination [1,4].

Surgical excision is the cornerstone of management for gastric teratomas and is usually curative. Because these tumors arise from the gastric wall (often the greater curvature), partial gastrectomy is frequently required to achieve clear margins [1,2]. Most patients-especially those with mature teratomas-are cured with surgery alone, with no malignant sequelae [3]. Even in immature cases, resection is typically sufficient, and adjuvant chemotherapy is reserved for the rare situation where a malignant germ cell component is identified [1]. Long‑term follow‑up is recommended, as late recurrences of gastric teratoma have been reported even decades after an initial “complete” resection [1,2].

Given the extreme rarity of gastric teratomas in neonates and the unique challenges illustrated by this tumor’s presentation, each new case report adds valuable insights to the literature. Here, we present a neonate with a high‑grade (immature) gastric teratoma involving the gastric wall that required complex surgical resection, underscoring the importance of considering gastric teratoma in the differential diagnosis of neonatal abdominal masses and highlighting the surgical and diagnostic nuances of this unusual tumor.

## Case presentation

A full‑term male neonate presented on the first day of life with abdominal distension and a firm mass palpable in the left upper quadrant. An initial abdominal radiograph demonstrated a subtle soft tissue fullness in the left upper quadrant with slight downward displacement of the bowel loops. The infant remained hemodynamically stable, tolerating minimal feeds, and was managed conservatively under close observation.

**Figure 1:**
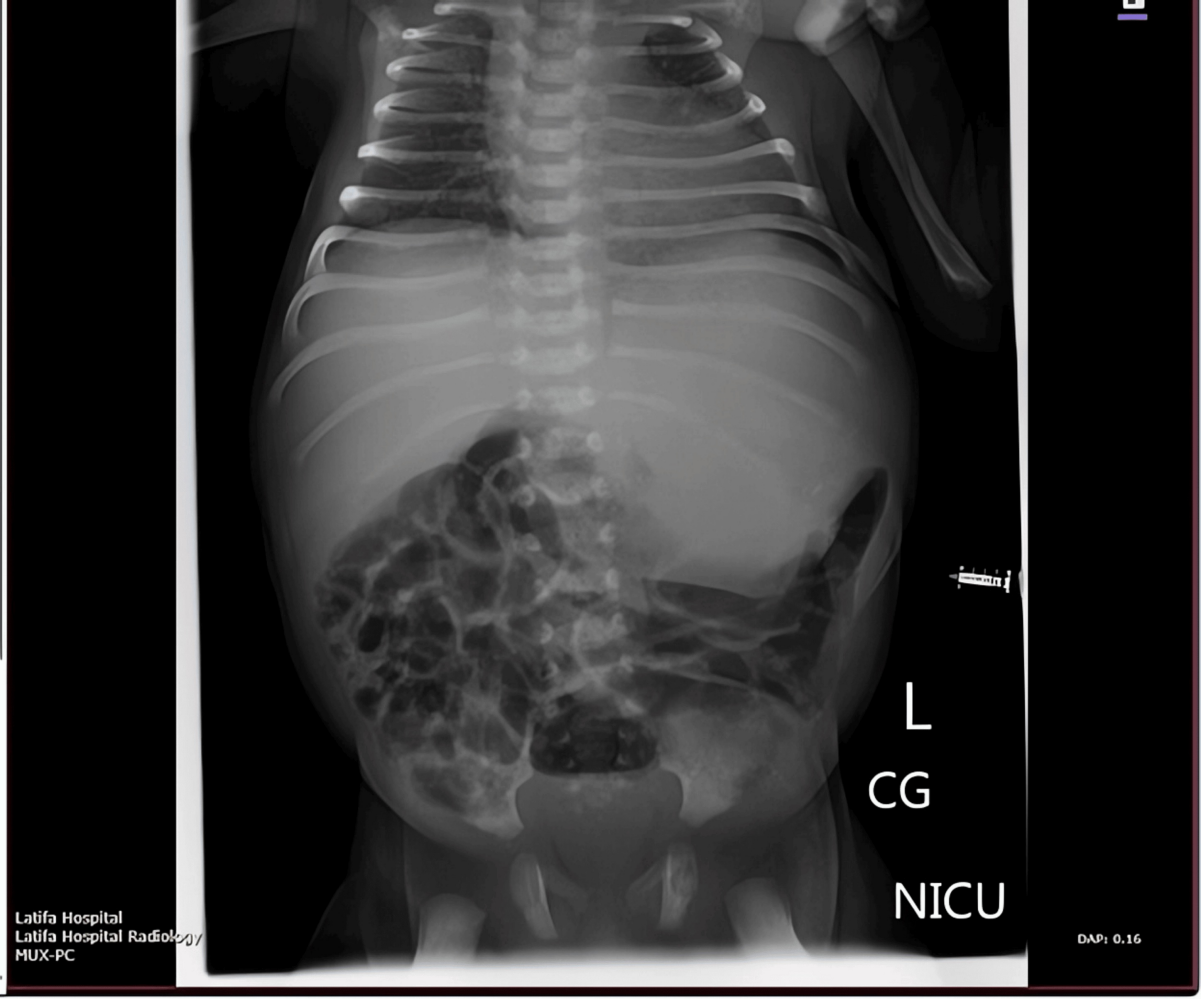
Day 8 radiograph showing emerging calcified epigastric mass Follow-up abdominal radiograph on day 8 of life, showing more defined gastric silhouette and progressive downward displacement of bowel loops. Coarse calcifications are visible overlying the mass in the left upper quadrant.

By the second week of life, the abdominal mass had become more pronounced. A follow‑up radiograph obtained on day 8 showed a more defined gastric outline in the upper abdomen with progressive inferior displacement of adjacent bowel loops (consistent with an enlarging mass effect). Notably, coarse calcifications were visible overlying the mass in the left upper quadrant, raising suspicion for a teratoma (Figure 1).

**Figure 2:**
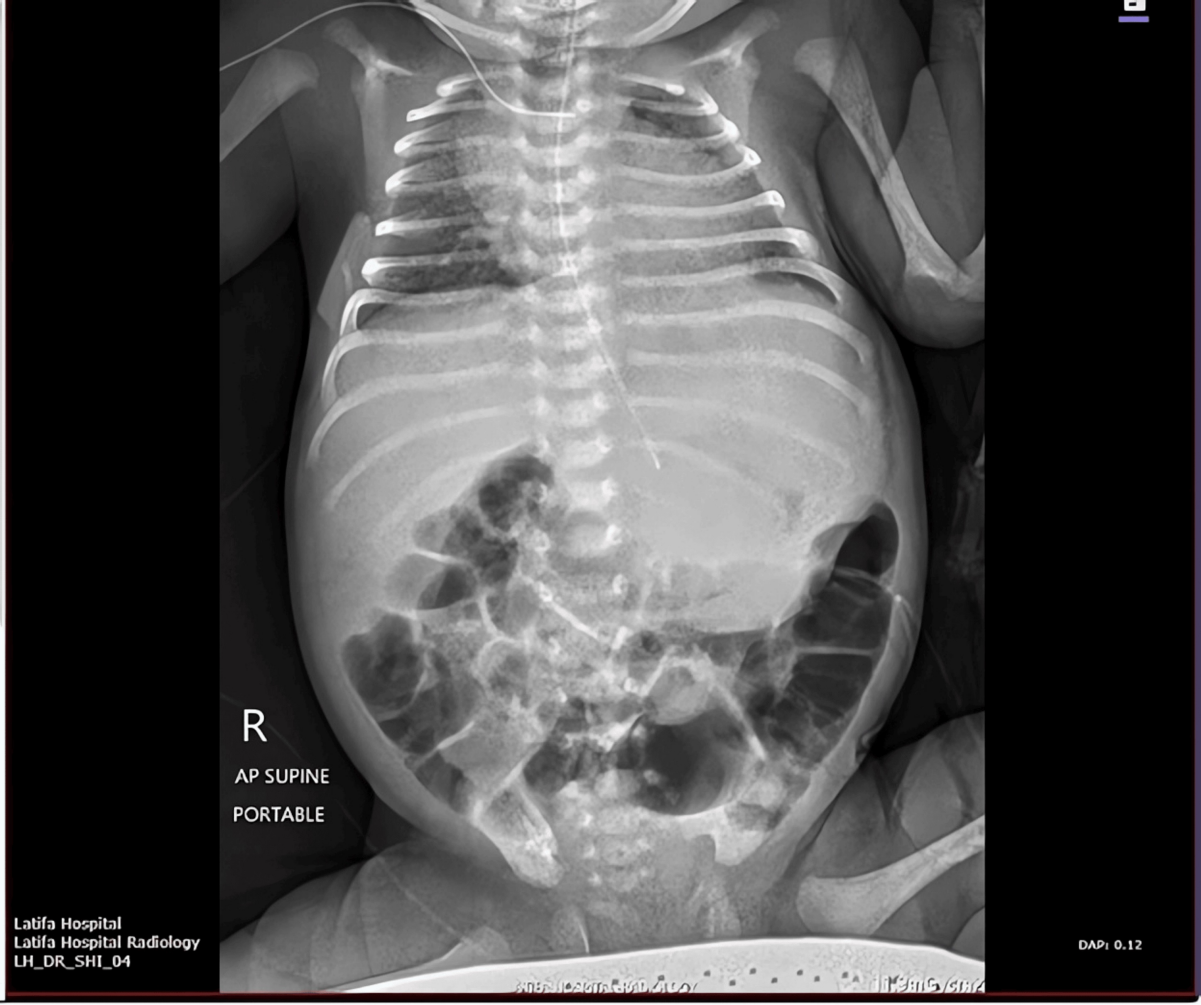
Day 11 radiograph demonstrating progressive enlargement of calcified epigastric mass Abdominal radiograph on day 11 demonstrating progressive enlargement of the epigastric mass and increasing calcific density within the lesion, with no evidence of bowel obstruction or free air.

An abdominal ultrasound was performed, revealing a mixed solid‑cystic mass arising from the stomach, with internal calcifications and well‑defined margins, further suggesting a gastric teratoma. Although the lesion continued to enlarge, the neonate remained clinically stable, allowing for thorough imaging and evaluation. Given the progressive enlargement of the lesion by day 11 of life (Figure 2) and the confirmation that the patient’s condition was still stable enough to proceed in a controlled fashion, the decision was made to proceed with surgical exploration.

**Figure 3:**
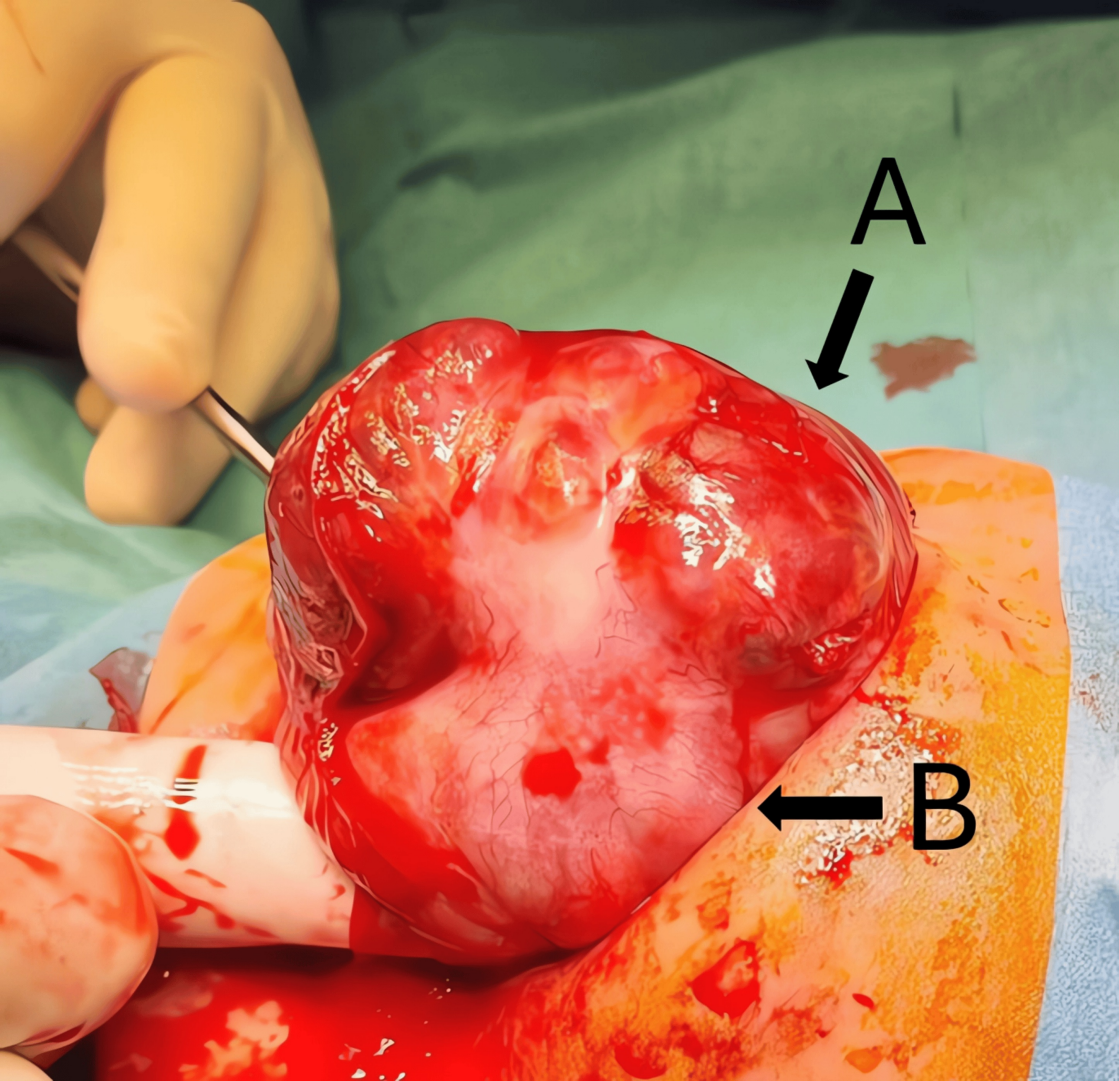
Intraoperative gastric teratoma before resection Intraoperative view showing a high-grade immature gastric teratoma (A) arising from the neonate’s stomach (B), prior to resection.

An exploratory laparotomy was performed on day 12 of life. Intraoperatively, a large lobulated tumor was found attached to the greater curvature of the stomach. The mass was predominantly exogastric, arising from the gastric wall without intraluminal extension. The tumor (A) was seen originating from the stomach wall (B) and was supplied by short gastric vessels (ligated during resection) (Figure 3).

**Figure 4:**
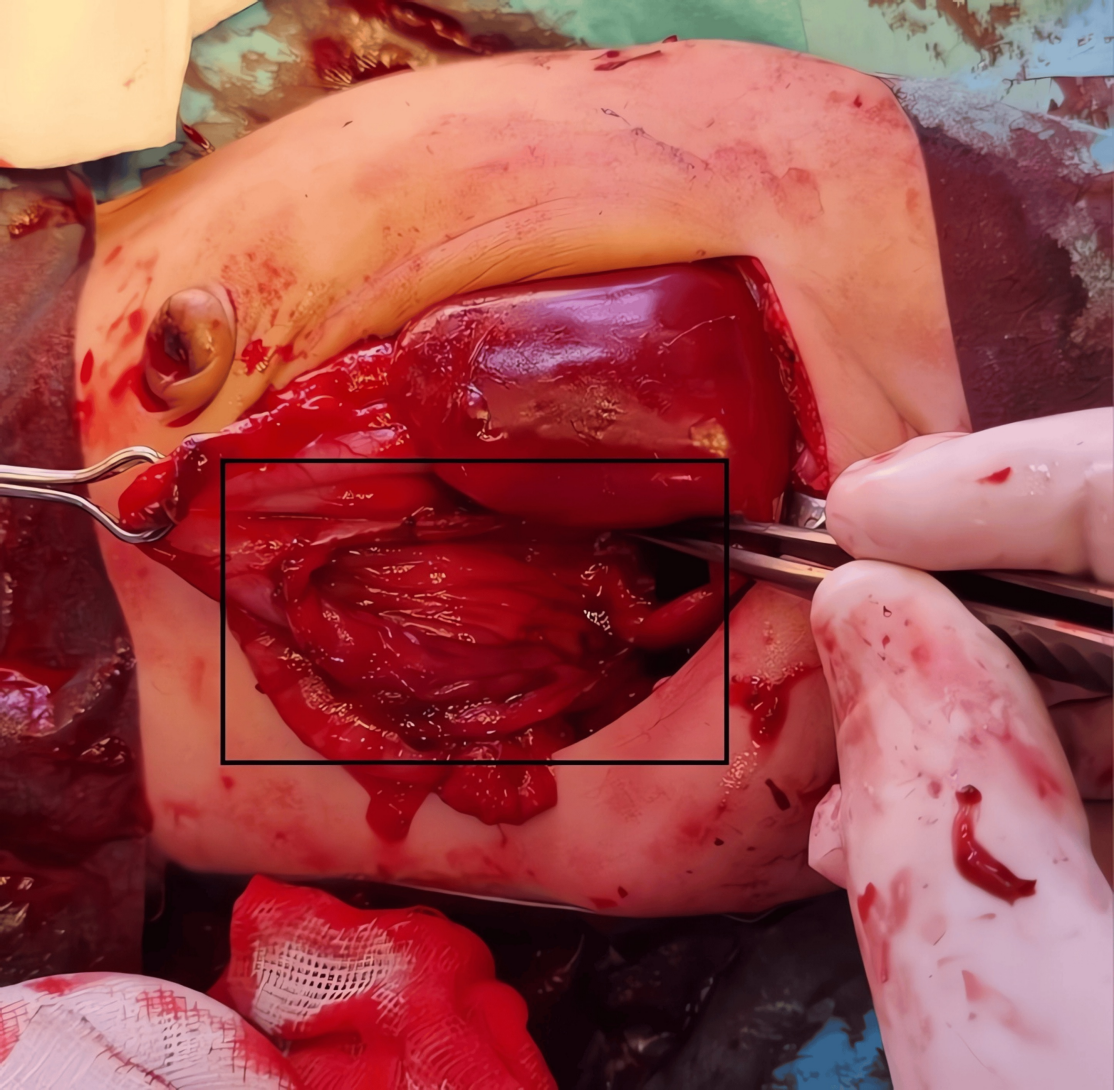
Post-excision defect in the gastric wall following teratoma resection

The tumor was carefully dissected free from the stomach and excised completely. This resection left a defect in the gastric wall approximately 3-4 cm in diameter on the greater curvature (Figure 4). No bleeding or additional masses were observed after tumor removal.

**Figure 5:**
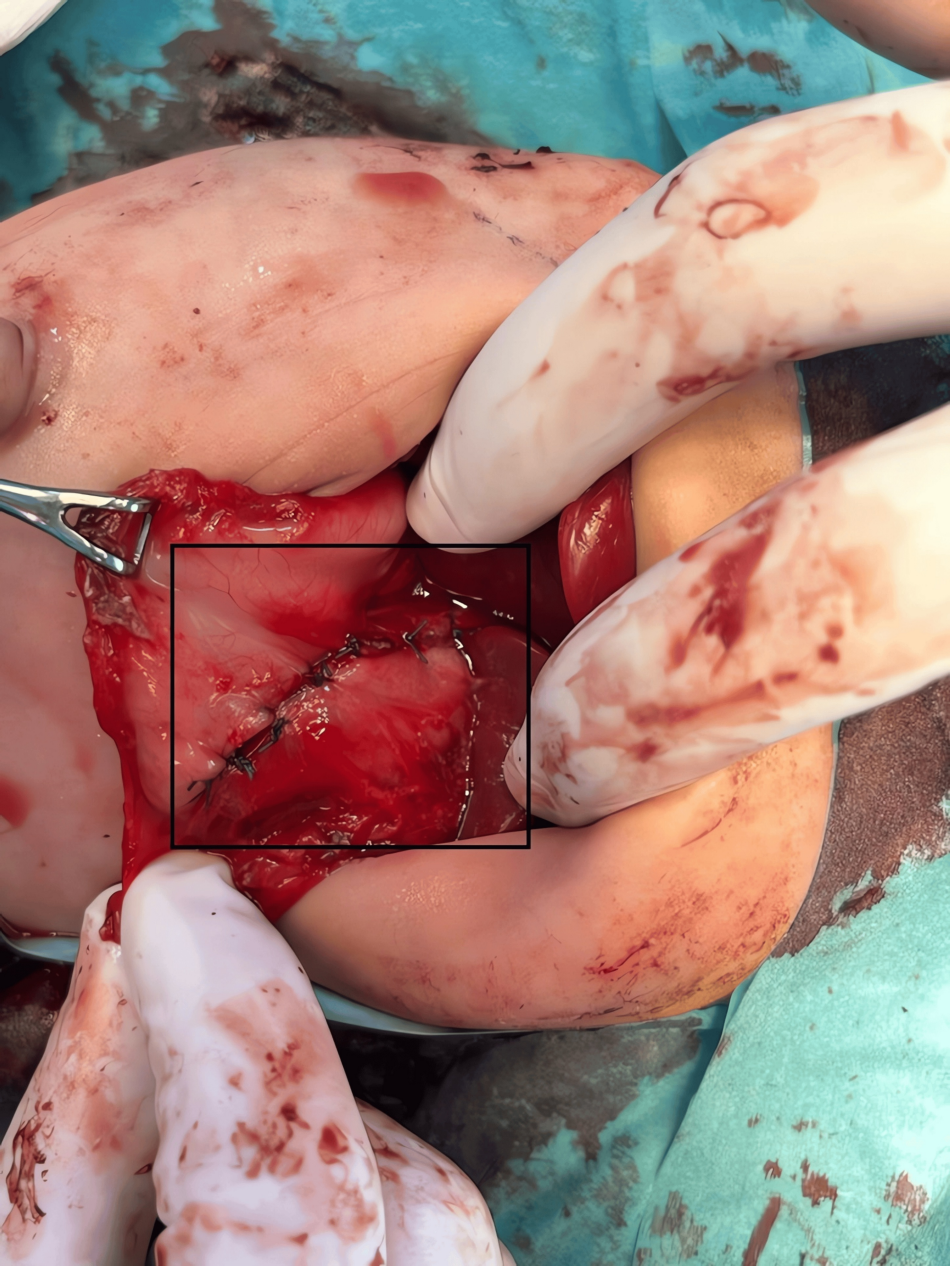
Primary closure of the gastric wall defect after teratoma resection

The gastric wall defect was then repaired with a two-layer closure using 3-0 Vicryl (Ethicon, Raritan, NJ, USA) absorbable sutures (Figure 5). The repair was checked intraoperatively and found to be tension-free and hemostatic. 

**Figure 6:**
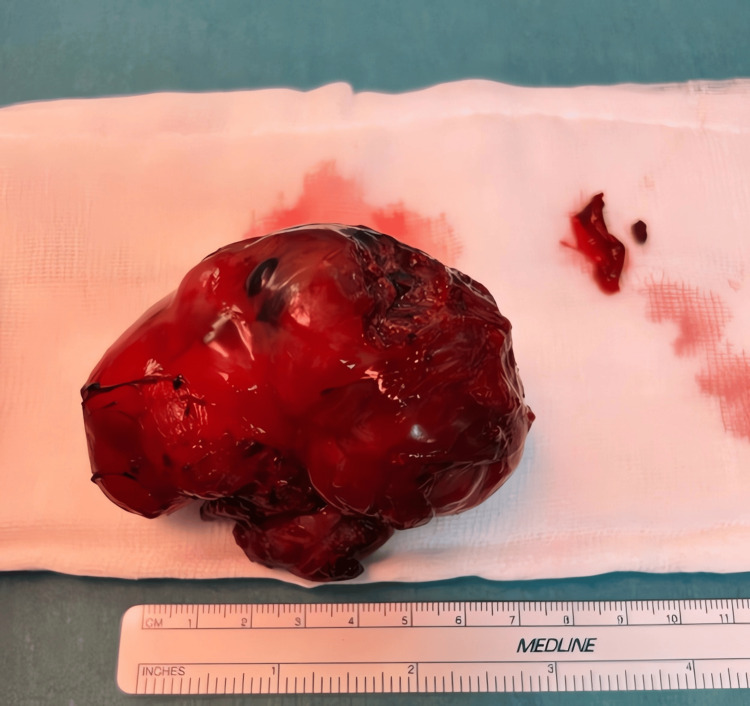
Gross specimen of the resected high-grade immature gastric teratoma

The resected specimen was a capsulated, fleshy mass measuring approximately 8×6×5 cm. On gross examination, it had a variegated red-gray cut surface with areas of cartilage and calcifications, consistent with a teratoma (Figure 6). There was no gross invasion into adjacent structures, and the resection margins appeared clear.

Histopathological analysis confirmed the diagnosis of high-grade immature gastric teratoma. Microscopic sections showed tissues from all three germ cell layers, including immature neural tissue (neuroectoderm) scattered among mature elements, indicative of Grade III immature teratoma. There was no malignant yolk sac (endodermal sinus) component identified. Given the complete surgical excision and the rarity of malignant behavior in gastric teratomas, no adjuvant chemotherapy was administered; however, the patient was scheduled for close surveillance with periodic abdominal ultrasounds and serial serum alpha-fetoprotein (AFP) levels.

**Figure 7:**
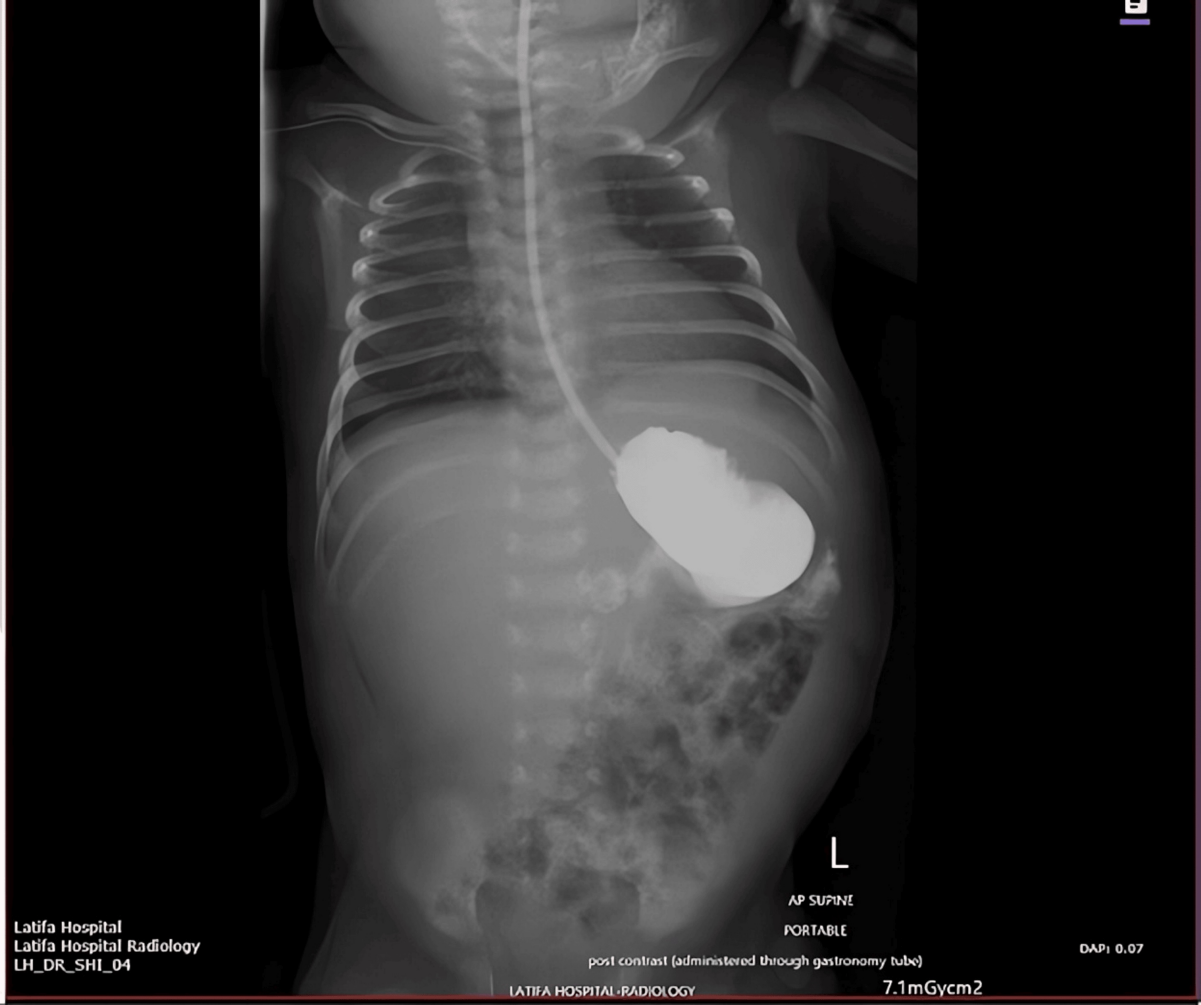
Postoperative contrast study on POD 11 (initial frame) showing intact gastric repair without contrast leak and nasogastric tube in situ POD: postoperative day

The infant’s postoperative course was uneventful. Enteral feedings were introduced gradually beginning on postoperative day (POD) 5, and tolerance was monitored closely. On POD 11, an upper gastrointestinal contrast study was obtained to assess the integrity of the gastric repair prior to advancing to full feeds. The initial contrast fluoroscopy frame demonstrated an intact gastric repair with no evidence of contrast leakage, and the nasogastric tube was seen in the proper position (Figure 7).

**Figure 8:**
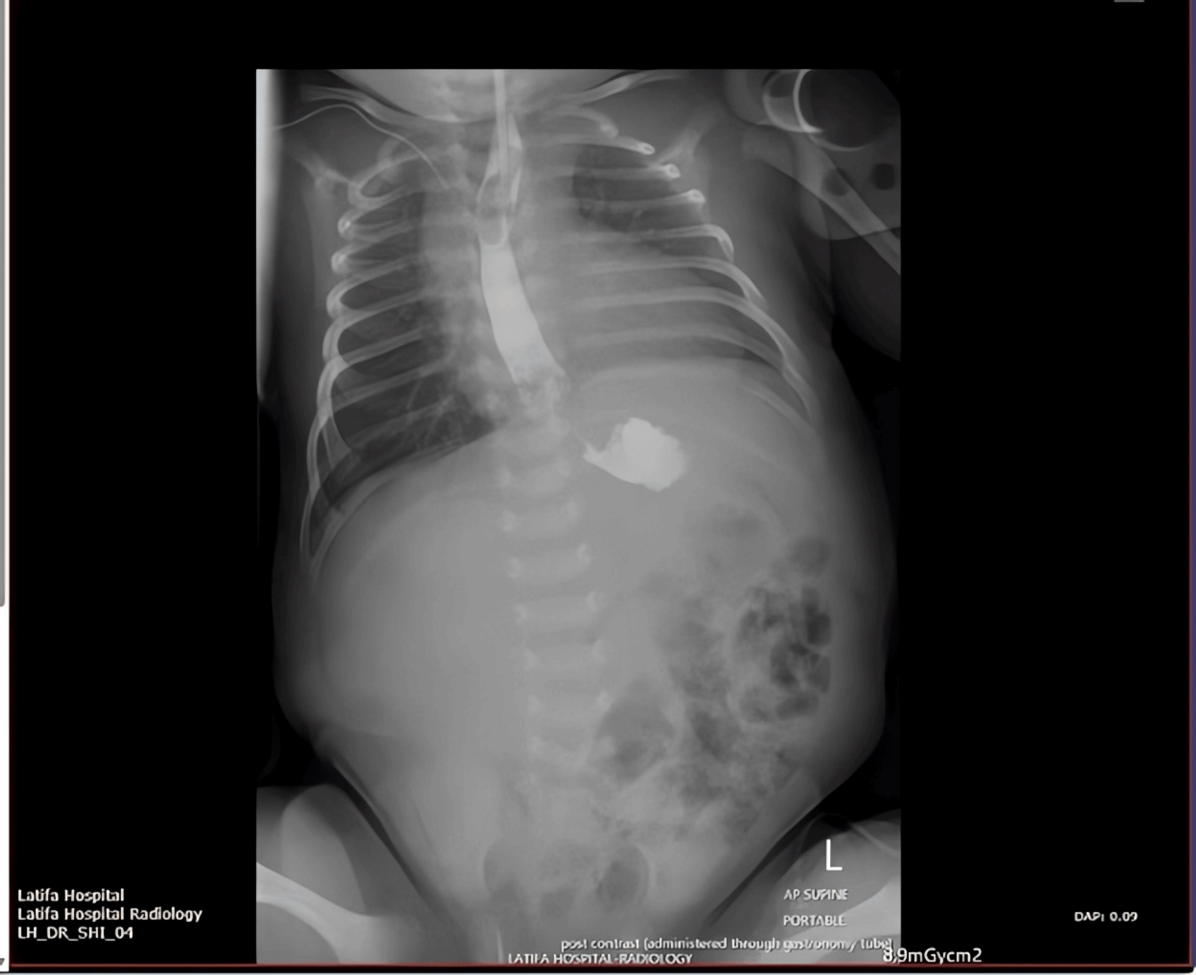
Postoperative contrast study (mid frame) showing contrast passage through the pylorus into the duodenum with no extravasation

A mid-sequence image showed contrast passing through the pylorus into the duodenum without any extravasation (Figure 8).

**Figure 9:**
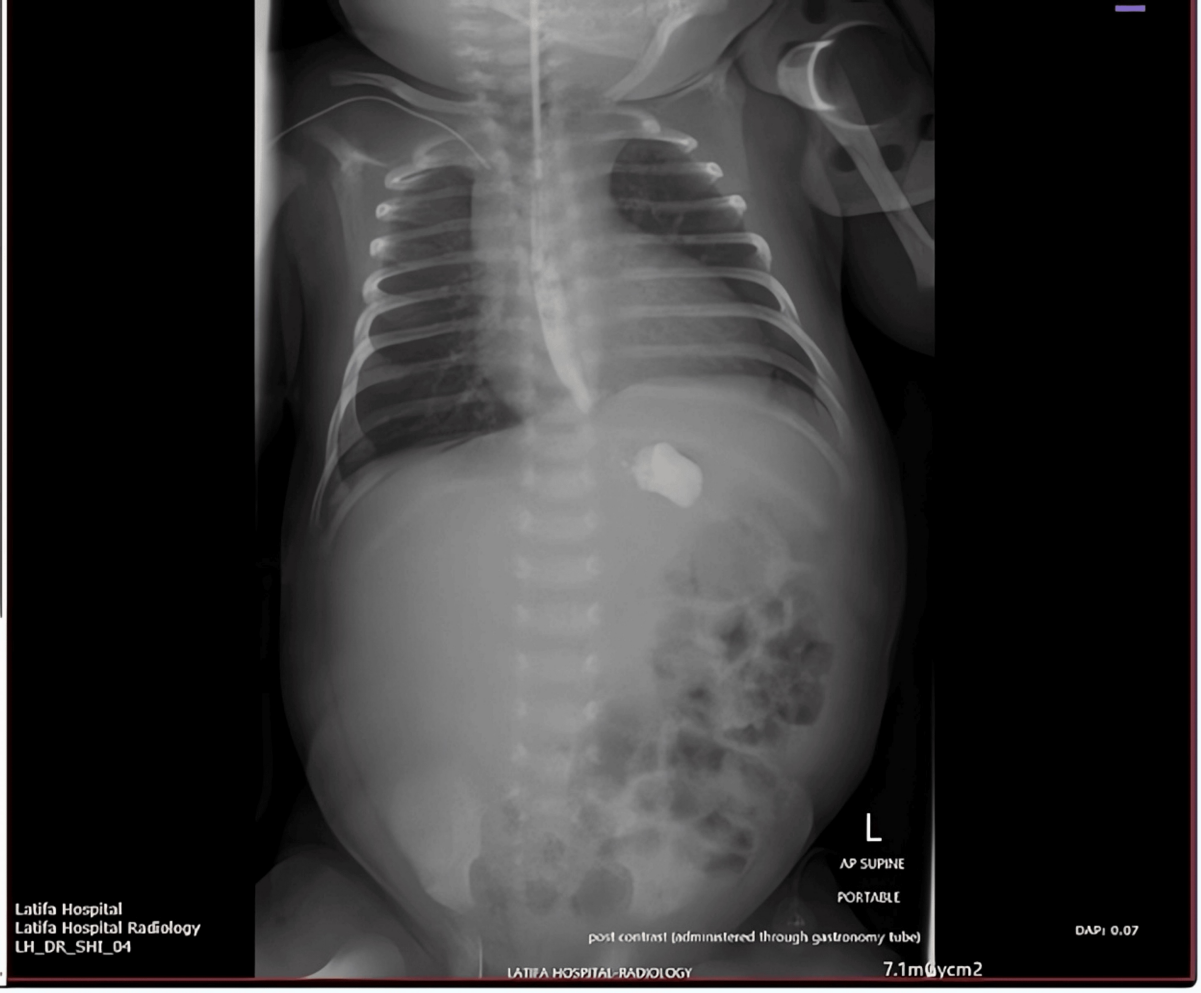
Postoperative contrast study (late frame) confirming complete gastric emptying and absence of contrast leak

By the late phase of the study, the stomach had completely emptied and no contrast leak was observed, confirming a successful repair (Figure 9).

After confirmation of normal gastric emptying, the infant’s feeds were advanced to goal volumes. He was discharged home in stable condition, tolerating full oral feeds. On follow-up at six months of age, the baby was thriving with no evidence of tumor recurrence and normal developmental progress.

## Discussion

Gastric teratomas represent less than 1% of all childhood teratomas [1], with a marked male predominance (over 90% of reported cases) [2]. The present case of a 10-day-old male individual with a high‑grade immature gastric teratoma underscores the unique diagnostic and treatment challenges of this entity in the neonatal period. Fewer than 200 cases have been documented in the literature since the first description in 1922 [5], and immature gastric teratomas comprise only a small fraction of these [1].

Diagnostic challenges

Preoperative diagnosis of gastric teratoma is challenging due to variable imaging appearances and overlap with more common neonatal abdominal masses [4,6]. Classically, these tumors appear as heterogeneous, mixed solid-cystic masses containing calcifications and fat, but these features may not be obvious in all cases [4]. For example, in one antenatally detected case, the lesion was initially presumed a meconium pseudocyst before MRI and postnatal studies established it as a gastric teratoma [6]. Similarly, Li and Wei reported a neonatal gastric teratoma misdiagnosed as a hemorrhagic lymphangioma due to its predominantly cystic nature [4]. The differential diagnosis for a neonatal abdominal mass is broad, including lymphatic malformations, neuroblastoma, and other germ cell tumors [6]. Ultimately, a definitive diagnosis requires histopathological examination.

Tumor markers

Tumor markers provide additional clues but must be interpreted cautiously in neonates. AFP is physiologically elevated at birth and declines over the first year of life; thus, a moderately high AFP may still fall within the normal neonatal range [1]. In contrast, a yolk sac tumor component typically produces AFP levels far above age‑adjusted norms and warrants further evaluation [7]. Persistently elevated or rising AFP beyond expected neonatal values should raise suspicion for malignant germ cell elements [7].

Surgical management

Complete surgical excision with clear margins is the cornerstone of gastric teratoma treatment and is generally curative [1]. Most tumors are exophytic, permitting wedge or partial gastrectomy with negative margins [2]. In this case, the tumor displayed both exogastric and endogastric components adherent to the liver, requiring careful dissection to achieve R0 resection. Ensuring microscopically negative margins is paramount, as residual tumor has been linked to recurrence [7].

Perioperative complications

Neonates undergoing major abdominal surgery are at risk for perioperative complications. Our patient experienced wound dehiscence and a thrombotic event, both managed successfully with secondary wound closure and anticoagulation. Although published series on gastric teratomas are limited, complications like wound breakdown or vascular thrombosis can occur in any extensive neonatal abdominal procedure [8]. Meticulous hemostasis, tension-free closure, and vigilant postoperative monitoring are essential strategies to prevent or promptly address these events.

Comparison with literature

The clinical spectrum of gastric teratomas ranges from asymptomatic abdominal swelling to life‑threatening neonatal distress. Most present with abdominal mass or distension and feeding difficulties [1]. Gastrointestinal bleeding, though uncommon, has been reported in cases with intragastric ulceration [4]. Prenatal presentations with hydrops fetalis and abdominal compartment syndrome have also been described [9]. Our patient’s presentation with a large abdominal mass in the first 10 days of life, yet without respiratory compromise, illustrates this variability.

Histopathology and prognosis

Histopathologically, gastric teratomas are classified as mature or immature. Mature teratomas, composed of well‑differentiated tissues from all germ layers, are benign, whereas immature teratomas contain embryonal elements and are graded according to the extent of neuroectodermal foci [1]. Our case, a Grade III immature teratoma, lacked a yolk sac or other malignant components, portending a favorable prognosis [1]. Thorough sampling and immunohistochemical evaluation are essential to exclude occult malignant foci [7].

Follow‑up recommendations

Long‑term outcomes are excellent when complete resection is achieved. Recurrences are rare but have been documented in immature cases, often within two years of surgery; hence, vigilant follow‑up with serial AFP and imaging is advised every three months for the first year, then biannually for two years, and annually thereafter for up to five to seven years [7]. Lifelong surveillance may be considered, given reports of late relapse decades after initial resection [10].

## Conclusions

This case underscores the importance of maintaining a high index of suspicion for gastric teratoma in neonates who present with an upper abdominal mass, especially when imaging reveals mixed solid-cystic components with calcifications or fat. Although mature gastric teratomas are more commonly encountered and typically follow a benign course, the presence of immature, high‑grade elements (as in this neonate) carries a potential for local aggressiveness and necessitates prompt, definitive management.

Complete surgical excision with clear microscopic margins remains the cornerstone of treatment and offers an excellent prognosis, even for high‑grade immature tumors, provided that thorough histopathological evaluation excludes malignant germ cell components. The perioperative challenges illustrated by this case, including vascular thrombosis and wound dehiscence, highlight the need for meticulous surgical technique, vigilant neonatal intensive care, and a multidisciplinary approach. Finally, diligent long‑term surveillance with serial imaging and tumor marker assessment is essential to detect the rare but important risk of recurrence. By sharing this experience, we aim to enrich the collective understanding of neonatal gastric teratomas and reinforce best practices in their diagnosis, surgical management, and follow‑up. Given the rarity of Grade III immature gastric teratomas, this report underscores the need for pediatric surgeons and neonatologists to consider this entity in their differential diagnosis and maintain meticulous perioperative management.
